# Influence of the chitosan morphology on the properties of acrylic cements and their biocompatibility†

**DOI:** 10.1039/d0ra06508k

**Published:** 2020-08-21

**Authors:** Sara Isabel Zamora Lagos, Jefferson Murillo Salas, Mayra Eliana Valencia Zapata, José Herminsul Mina Hernandez, Carlos Humberto Valencia, Luis Rojo, Carlos David Grande Tovar

**Affiliations:** Escuela de Ingeniería de Materiales, Facultad de Ingeniería, Universidad del Valle Calle 13 No. 100-00 Santiago de Cali 760032 Colombia sara.zamora@correounivalle.edu.co Jefferson.murillo@correounivalle.edu.co valencia.mayra@correounivalle.edu.co jose.mina@correounivalle.edu.co; Escuela de Odontología, Grupo biomateriales dentales, Universidad del Valle Calle 13 No. 100-00 Santiago de Cali Colombia carlos.humberto.valencia@correounivalle.edu.co; Consorcio Centro de Investigación Bioméedica en red, CIBER-BBN Madrid 28029 Spain; Instituto de Ciencia y Tecnología de Polímeros, Consejo Superior de Investigaciones Científicas Madrid 28006 Spain rojodelolmo@ictp.csic.es; Programa de Química, Facultad de Ciencias, Universidad del Atlántico Carrera 30 Número 8-49 Puerto Colombia 081008 Colombia carlosgrande@mail.uniatlantico.edu.co

## Abstract

Acrylic bone cements (ABC) are materials widely used in orthopedics and biomedical applications. Several active compounds have been introduced to ABC formulations to improve their mechanical properties and bifunctionality. In this research, we studied the effect of the addition of chitosan (CS) microspheres and chitosan sheets on ABC formulations. For mechanical performance optimization, the compression strength was taken as a response variable using an extreme vertices mixing design with fraction by weight of CS and poly(methyl methacrylate) (PMMA) as the variable factors. According to the statistical analysis, the control samples (without CS), samples with 7% (wt.) of CS sheets, and samples with 17% (wt.) of CS spheres presented the best compression properties: 90.6 MPa and 95.6 MPa, respectively. The study of these formulations confirmed that CS spheres allow a higher amount of loading on the formulation, maintaining comparable compression strength. By ^1^H-NMR, it was observed that the residual monomer was similar in all wording. The hydrolytic degradation assay in simulated body fluid (SBF) determined that the sphere incorporation increased by 50% and 35% for the water uptake and weight loss values, respectively, when compared with the reported values with CS sheets. By morphological analysis *via* SEM, it was observed that the porosity increased considerably in the presence of CS spheres throughout the immersion time in SBF. The subdermal implant results demonstrated excellent compatibility between the cement studied and the biological environment.

## Abstract 2

Acrylic bone cements (ABC) are materials widely used in orthopedics and biomedical applications.

## Graphical abstract

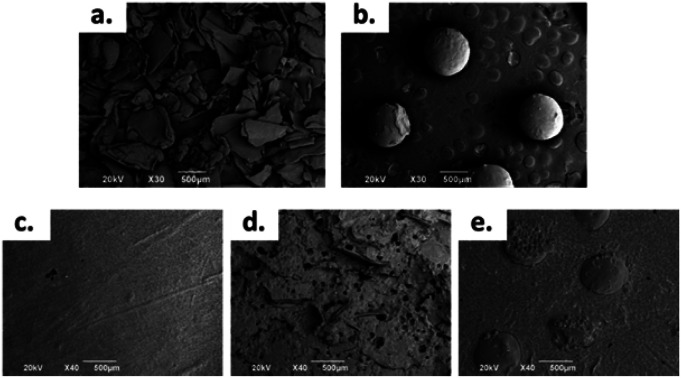

## Introduction

Acrylic bone cements (ABC) have been used clinically for many years in orthopaedic surgery for the fixation of artificial joints with promising results.^1^ The main function of bone cement is to transfer strains from the prosthesis to the bone, and increase the load-sharing capacity of the prosthesis.^2^ Cements must endure considerable stresses, and thus fulfilling the standardised mechanical properties are important for its clinical success.^3^

ABCs used in surgical applications are relatively weak in terms of their tensile strength, but they are resistant to compression.^4,5^ The compression strength values are in the range of 85–114 MPa, which are related to 50–70% of the cortical bone strength.^6^ However, despite the good results of implant fixation with bone cements, they still show some clinical disadvantages such as inert nature, which eventually generates aseptic loss or prosthesis deterioration.^7^ Currently, different approaches are still under investigation to overcome these limitations. Biocompatibility and functionality improvement of cements have been performed by inducing porosity with vacuum mixing devices,^8,9^ or adding bioactive compounds in the curing formulation such as nano-hydroxyapatite-based fillers.^10^ The bactericidal capacity was introduced using essential oil-based monomers,^11^ graphene oxide, graphene,^12^ and chitosan (CS) polymers. Chitosan has excellent mucoadhesive, hemocompatibility, biodegradability, and is not toxic. Chitosan also has anti-tumour, antioxidant, and antimicrobial properties, so it has become a very attractive biomaterial for different applications in biomedicine.^13–15^

Cement formulations, including CS, demonstrated physical and biological improvements.^16,17^ Several studies on the addition of CS sheets to acrylic bone cement showed a positive impact on the reduction of the maximum curing temperature and biocompatibility, with no significant effects on the tensile properties.^8^

On the other hand, Shi *et al.* investigated the use of CS nanoparticles (CS NP) as bactericidal agents in PMMA bone cement against Gram-positive strains, such as *S. aureus* and *S. epidermidis*, after a prolonged period of time.^18^ In addition, Meng *et al.* analysed the effects of resorbable CS microspheres into calcium phosphate cement formulations, improving their mechanical behaviour and inducing a porous morphology on the cements after immersion in simulated biological fluid (SBF) with a healthy *in vivo* recovery process.^19^ However, very few formulated cements have reported the effect of the CS morphology in the final properties of the material, like curing parameters, mechanical properties, or *in vitro* responses.

The purpose of the present study was to analyse the effect of adding CS in two different morphologies (sheets and microspheres) on the mechanical, physical and biological properties of ABCs by analysing the residual monomer percentage, the mechanical properties and histological results of ABCs implanted in the subdermal tissue of Wistar rats for 30 days.

## Materials and methods

### Materials

The solid phase of ABCs was composed of PMMA microparticles acquired from New Stetic S.A. (Medellin, Colombia), barium sulphate (BaSO_4_) from Alfa Aesar (Tewksbury, MA, USA), and benzoyl peroxide (BPO). The chitosan sheets were from shrimp shells (*M*_w_ = 190 000–310 000 and a deacetylation degree of 88%) from Sigma-Aldrich, with CAS number 9012-76-4 and MDL number MFCD00161512 (Palo Alto, CA, USA). CS spheres with a maximum diameter of 1 mm were obtained from the transformation of chitosan sheets by the coacervation method based on the methodology reported by Dias and Queiroz.^20^ CS spheres were also used in the preparation of the solid phase.

The liquid phase was composed of methyl methacrylate (MMA), 2-(diethylamino)ethyl acrylate (DEAEA), 2-(diethylamino)ethyl methacrylate (DEAEM) (Sigma-Aldrich, Palo Alto, CA, USA), and *N*,*N*-dimethyl *p*-toluidine (DMPT) (Merck, Burlington, MA, USA), according to Table 1. All materials were used as received from the supplier, except for BPO, which was recrystallized from methanol.

Formulation of acrylic bone cement

**Table:** 

Solid phase	Amount (wt%)
PMMA	88–68
BaSO_4_	10
BPO	2
CS^a^	0, 7.64 or 17^a^

a CS was added to the solid phase as sheets (7.64 wt%) or spheres (17 wt%). Control samples without CS (0 wt%) were used as the reference group.

**Table:** 

Liquid phase	Amount (wt%)
MMA	97.5
(Co-monomer 1) 2-(diethylamine)ethyl methacrylate	1.25
(Co-monomer 2) 2-(diethylamine)ethyl acrylate	1.25

### Mixture design

The composition of experimental formulations used in this study were designed based on the mixing multi-component methodology (extreme vertices) previously reported for composites mixtures.^21^ In this case, the effect of the CS morphology added to the bone cement was studied, including two designs of mixtures of independent extreme vertices for each morphology. The following components were used for sheet design: PMMA and CS, with a process variable identified as size; and for the design of spheres, the components PMMA and CS without process variable.

### Response optimization

The intervals for the experimental region of each model are given in Table 1 with compression strength as the response variable for both experimental designs.

Experimental components were used in a regression model with linear effects and significant interaction, which is represented by eqn (1):^22^

1 *y* = *β*_0_ + *β*_1_*x*_1_ + *β*_2_*x*_2_ + *β*_3_*x*_1_*x*_2_ + *ε*

where *Y* is the response variable (compression strength), and *e*_*i*_ is the error in the *u*-exempt test. The parameter *β*_*i*_ represents the effect of the *i*-exempt pure component, *β*_*ij*_ is the impact of the interaction between components *i* and *j*, and *β*_*ijk*_ is the implication of the triple interaction components *i*, *j*, and *k*. The *e*_*i*_ errors are assumed to be independent and identically distributed usually with mean *μ* = 0 and variance *σ*^2^. The parameters *β*_*i*_, *β*_*ij*_, *β*_*ijk*_ can be estimated by least-squares.

Tables S1 and S2 (ESI†) present the different proportions of each component obtained under the design of extreme vertices mixtures for sheets and spheres, respectively.

The design experiment optimization included the analysis of each optimization graph thrown by the MINITAB 18 program in the final vertices experiment designs for sheets and spheres.

The conditions for the compression strength were as follows: lower limit = 44 MPa, target = 90 MPa and upper limit = 100 MPa for the sheets design, and lower limit = 85 MPa, target = 95 MPa, and upper limit = 100 MPa for the sphere design.

### Bone cement preparation

Each component proportion was considered in the designs of the sheet and sphere experiments for the acrylic bone cement preparation. The cement preparation consisted of a solid–liquid phase ratio of 2 : 1 (Table 1). Both solid phase and liquid phase were weighted separately and manually mixed according to ISO 5833.^23^

### Physicochemical characterization of bone cement

#### Particle morphology and surface microstructure

The morphology of the CS sheets and spheres, and the bone cement surfaces were studied by scanning electron microscopy (SEM, JEOL JSM-6490LA, Musashino, Tokyo, Japan). Samples were gold sputtered before examination under an acceleration voltage of 20 kV.

### Thermal characterization

Thermogravimetric analysis (TGA, TA Instruments Q50, United States) technique was used to study the thermal degradation of the control and experimental bone cements. The heating ramp used was 20 °C min^−1^ from room temperature to 550 °C under N_2_ atmosphere.

### Mechanical characterization

Mechanical compression and flexion tests were performed following ISO 5833 for acrylic bone cement.^23^ The samples were prepared by compression moulding with a hydraulic press (Carver 4389, United States) of 1000 psi at room temperature, and the Teflon mould dimensions were defined by the standard.

#### Compression test

According to ISO 5833, 5 cylindrical specimens of each formulation of ABCs were tested 24 hours after forming. The test was performed with a universal testing machine (Tinius Olsen-H50KS, USA) at a head speed of 20 mm min^−1^ with a load cell of 50 kN.

#### Flexural test

According to ISO 5833, 5 rectangular specimens of each formulation of ABCs were tested 24 hours after preparation. The four-point flexion test was performed with a universal testing machine (Tinius Olsen-H50KS, USA) at a 5 mm min^−1^ head speed with a cell load of 10 kN, and a distance between internal supports of 20 mm and external supports of 60 mm. The bending module and strength values were calculated according to the international standard.^23^

### Hydrolytic degradation assay

ABC immersion for two months in simulated body fluid (SBF) prepared according to the Kokubo and Takadama method was used to evaluate hydrolytic degradation.^24^ SBF was stored in plastic bottles at a temperature between 5 and 10 °C, and used within less than one month. During immersion, the pH of the solutions, absorption, and weight loss of the samples were analysed.

According to ASTM F 1635, 5 samples of each formulation of ABCs were incubated at 37 °C for 24 hours before SBF immersion, and stored in an incubator (Memmert IN 110, Germany) at 37 °C. The buffer was replaced every week, except for the pH variation determination tests. Absorption and weight loss were calculated from water uptake (eqn (2)) and weight loss (eqn (3)), respectively:2
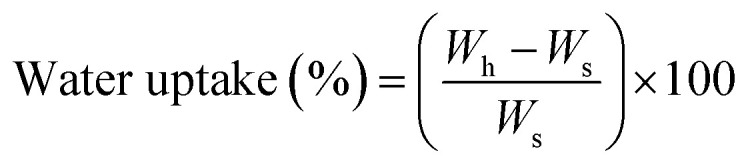
3
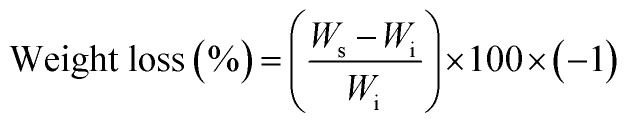
where *W*_h_ is the wet weight of the sample, *W*_s_ is the dry weight of the sample after SBF immersion, and *W*_i_ is the initial weight of the sample.

### *In vivo* biomodel assay

An *in vivo* biomodel assay was performed to study the compatibility between the cements and the biomodel tissues. CS cement samples with different morphologies (three replicates per formulation) and a BioMend® commercial porcine collagen as a control sample (ZIMMER BIOMET, Miami, FL, USA) were implanted. 3 samples of each formulation of ABCs of 10 mm diameter and 2 mm thickness were implanted in the subdermal tissue of three adult Wistar rats. The preparations consisted of a dorsal incision in accordance with the ISO 10993-6 standard.^25^

After 30 days of implantation, the samples were fixed in buffered formalin, dehydrated in ascending concentration alcohol solutions (70%, 80%, 95% and 100%), diaphanized with xylol and infiltrated with paraffin. After that, the samples were cut at 4 μm using a Thermo Scientific™ Histoplast Paraffin™ and an Autotechnicon Tissue Processor™ (Leica Microsystems, Mannheim, Germany).

Hematoxylin and eosin technique and Masson's trichromacy technique were used for histological analysis of the samples. Image analysis was performed with a Leica DM 750 microscope containing a Leica DFC 295 camera, and Leica Application Suite version 4.12.0 (Leica Microsystems, Mannheim, Germany) was used. The macroscopic images were taken with a Samsung Dv150f Used 16 mp digital camera. For SEM interpretation, samples were dehydrated in ascending alcohol concentration (70%, 80%, 95% and 100%). Finally, to add conductivity, they were coated in a copper bath and analysed with a scanning electron microscope (JEOL JSM-6490LA, Musashino, Tokyo, Japan).

All animal procedures were performed in accordance with the Guidelines for Care and Use of Laboratory Animals of the Faculty of Medical Sciences of the Universidad del Valle, and approved by the Animal Ethics Committee of the Universidad del Valle by means of the CEAS 001-016 certificate.

## Results and discussion

### Optimization of the loading amount of PMMA and CS was necessary for each experimental design model

In the optimization charts (Tables S1 and S2,† notes), the best combination of input variables (X1: PMMA, X2: CS) was assessed, and an additional process variable was selected for the case of sheet design (size 1). Initially, the ratio of PMMA and CS was evaluated to maximize the compress strength, corresponding to 88% PMMA and 0% CS, which resulted in a strength of 107 MPa for both designs (Fig. S1, S2 and S3†).

Optimization charts offer the ability to modify factor values conveniently. In the sheet design, by increasing the CS content to 0.0764 with a fraction of 0.8036 PMMA, the model predicted a compression strength of 90.60 MPa with the predictability of D-1 (Fig. S3, ESI file†). On the other hand, in the case of the sphere design, the increase in CS content to 0.17 CS and a fraction of 0.71 PMMA predicted a compression strength of 95.65 MPa with the predictability of D-1 (Fig. S4, ESI file†). The compression strength values obtained are higher than the minimum required by ISO, which is 70 MPa.^23^

Chitosan itself has low mechanical strength, so when added to an acrylic matrix, mechanical strength will inevitably be compromised.^26^

**Fig. 1:**
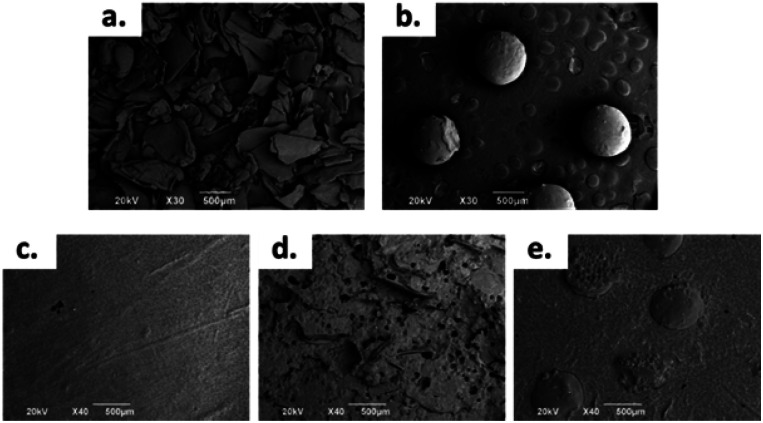
SEM images at 30×: (a) CS Sheets, (b) CS spheres, and bone cement images at 40×: (c) control sample, (d) sample with 7 wt% of CS sheets, (e) sample with 17 wt% of CS spheres.

However, when working with chitosan sheets, the mechanical behaviour is drastically affected due to the angular morphology of the particles. In addition, these have a smaller particle size than CS spheres (Fig. 1a and b). Thus, it is possible for the CS sheets to disperse in a more homogeneous way, so there will be more points acting as a stress concentrator.^17,27^ When the CS spheres have a regular morphology and a particle size that is greater than 1 mm, it is possible to increase the amount of CS load without drastically compromising the mechanical behaviour with these characteristics. However, there is a maximum amount of CS spheres to add. If too many spheres are added, the matrix could be split apart by the presence of spheres, decreasing the mechanical properties.^19^

Therefore, the formulations for working were the control (without CS), a sample with 7 wt% of CS sheets and a sample with 17 wt% of CS spheres.

### Morphology studies

The morphology of the CS sheets, CS spheres and bone cements are shown in Fig. 1. The irregular and angular shape of the CS sheets (Fig. 1a) and the regular shape of the spheres (Fig. 1b) can be appreciated. Introduction of CS into the cement bulk increased the porosity of the PMMA matrix, especially for the CS sheets (Fig. 1d) with an average pore size of 70–80 μm. Cement with CS sheets presented a homogeneous distribution (Fig. 1c), when analysed by SEM. The cement with CS spheres also showed good dispersion inside the PMMA matrix, together with small gaps around the spheres (Fig. 1d). According to Tan *et al.*, the CS particles have a weak interaction capacity with the PMMA cement matrix, supported by hydrophobic physic interactions. For this reason, gaps can be formed between the CS particle and the polymer matrix, favouring the porosity of cement during the setting process.^16^

### Thermal characterization

**Fig. 2:**
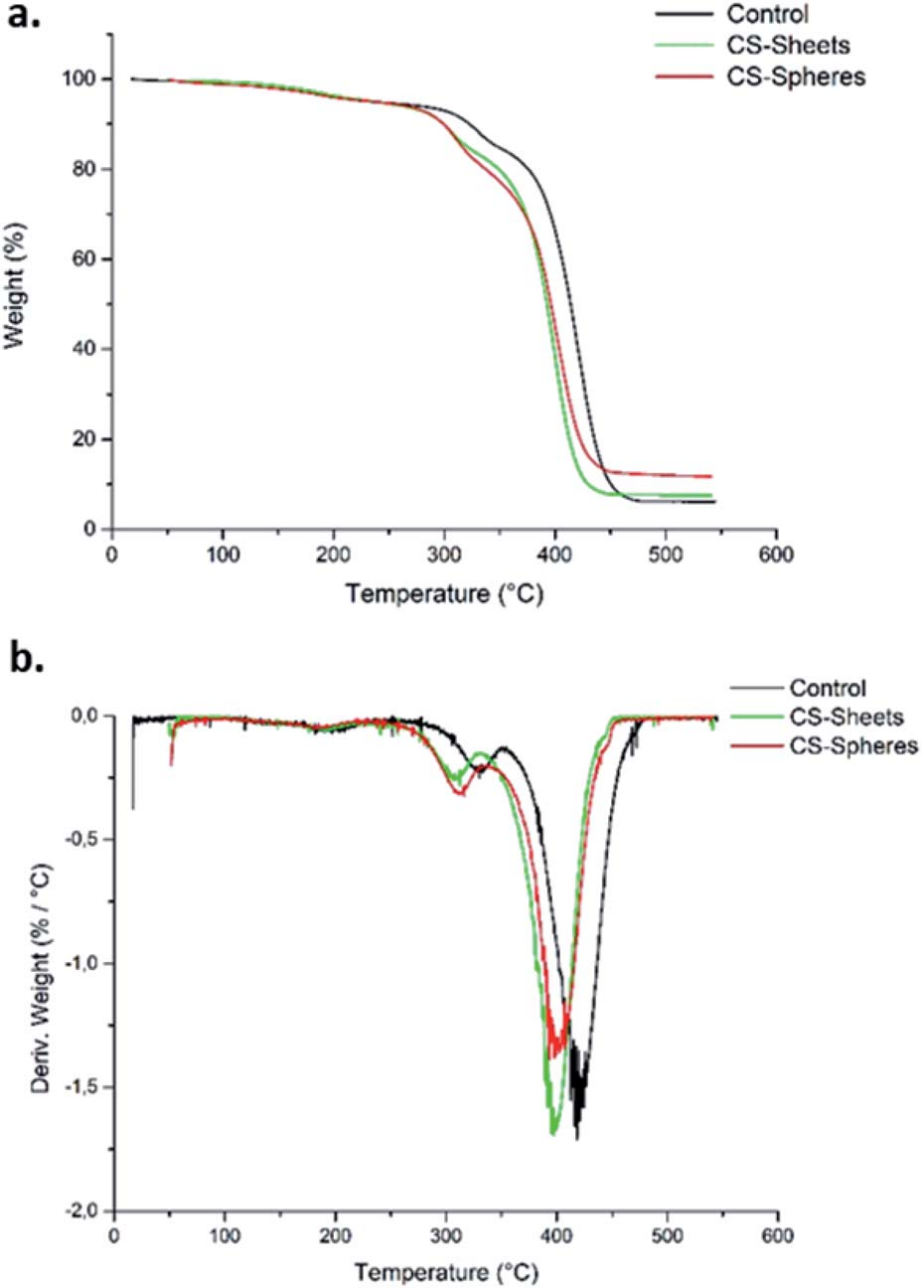
Thermal analysis recorded for bone cements. (a) TGA curves, (b) DTGA curves.

Fig. 2a shows the TGA curves for the control samples (without CS), samples formulated with sheets, and samples formulated with CS spheres. There was a weight loss of 5% around 250 °C. This weight loss corresponds to the desorption of physically adsorbed water on the sample surfaces.^28^ After this temperature, the weight loss curves become increasingly pronounced up to a temperature of 450 °C for samples with CS sheets and spheres, and at 470 °C for the control sample. Therefore, it can be said that CS decreases the thermal stability of the bone cements. Other authors have reported similar thermal behaviour by adding chitosan to ABCs.^17^

Besides, according to the DTGA curves (Fig. 2b), the presence of CS in the bulk induces a decrease in temperature for the maximum rate of decomposition (*T*_max_) from 418 °C for the control cement to 397 °C and 399 °C for the CS sheets and CS spheres, respectively. Previous studies indicate similar behaviour relative to the control sample.^29^

This thermal behaviour can be explained by the fact that PMMA loses stability at high temperatures of around 330 °C, which leads to depolymerization by chain scission at the same temperature range where CS decomposes. This produces volatile compounds, and thus accelerates the bulk decomposition and affects the thermal stability.^28,30^

### Mechanical characterization

The mechanical properties of bone cement were evaluated using compression and bending strength measurements.

#### Compressive test

The results of the compressive strength of experimental bone cement are shown in Table 2. The values obtained from the compression strength are in agreement of ISO 5833 standard (>70 MPa).^23^ The compressive strengths obtained were 108.68 ± 1.11 MPa, 94.08 ± 1.78 MPa and 93.32 ± 1.93 MPa for the control, sample with CS sheets and sample with spheres, respectively. By adding 17 wt% of CS spheres, a strengthening in compression behaviour was obtained for cements similar to that obtained by adding 7 wt% of CS sheets. This behavior can be explained because the CS spheres have a regular morphology unlike CS sheets, which are more irregular. Therefore, CS sheets reduce the mechanical properties because the edged flake shape facilitates the propagation of cracking with a cumulative effect of the effort.^17^

**Table:** Compression and flexural modulus values ± standard deviation obtained for experimental bone cements. Control (sample without CS), (sheets) sample with 7 wt% of CS sheets, (spheres) sample with 17 wt% of CS spheres

Sample	Compressive strength (MPa)	Flexural strength (MPa)	Flexural modulus (MPa)
Control	108.68 ± 1.1	50.97 ± 2.1	2422.83 ± 215.9
Sheets	94.08 ± 1.8	34.25 ± 0.76	2113.76 ± 375.5
Spheres	93.32 ± 1.9	26.34 ± 0.72	2068.23 ± 447.1

#### Flexural strength test

Flexural strength values recorded for experimental bone cements are shown in Table 2. Samples containing CS did not reach the minimum value required by the ISO 5833 (>50 MPa). However, the recorded flexion module did meet the requirements (>1800 MPa).^23^ It was observed that the presence of CS in the cement formulation resulted in a reduction of 47% in the flexural strength when incorporated in the form of spheres, and almost 31% when included in the form of sheets. A similar behavior occurs when analyzing values obtained from the flexural module. The results between the cements demonstrated that the differences in the flexural strength are low, as compared to the differences in the CS content (7 wt% for CS sheets and 17 wt% CS spheres, respectively).

According to the mechanical characterization, the addition of a higher amount of CS spheres is possible without affecting the mechanical properties.

### Hydrolytic degradation evaluation

**Fig. 3:**
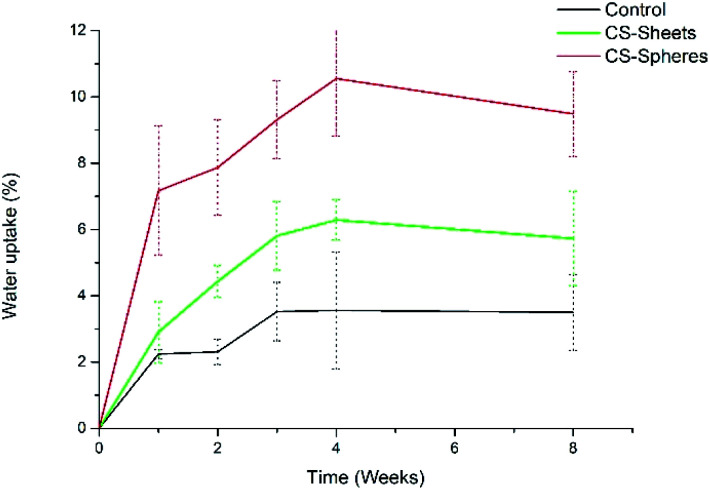
Water absorption for experimental bone cements: control, cements with 7 wt% of CS sheets, and cements with 17 wt% of CS spheres. Collection time-points are represented by dots and standard deviation.

A water absorption and hydrolytic degradation test was performed to determine the behaviour of experimental cements in SBF, which contained ion concentrations almost equal to those of human blood plasma.^24^Fig. 3 shows the water uptake capacity registered for the control and CS containing cements over time. Cements with CS spheres showed a higher absorption capacity than CS sheet samples, and CS sheets-based cements were likewise higher than the control group. This trend can be explained due to the presence of CS increasing the hydrophilicity of the bulk and partial solubility in SBF, facilitating the formation of interconnected pores and the entry of SBF.^9^ Samples with CS spheres have a higher absorption capacity because their CS weight content is higher (17%), with a superior contact surface than the content of samples containing sheets (7%). Absorption values found at week eight of immersion were 4%, 6%, and 9% for the control samples, sheets and spheres, respectively, in accordance to other authors reporting similar water absorption values for conventional acrylic cement.^17^

**Fig. 4:**
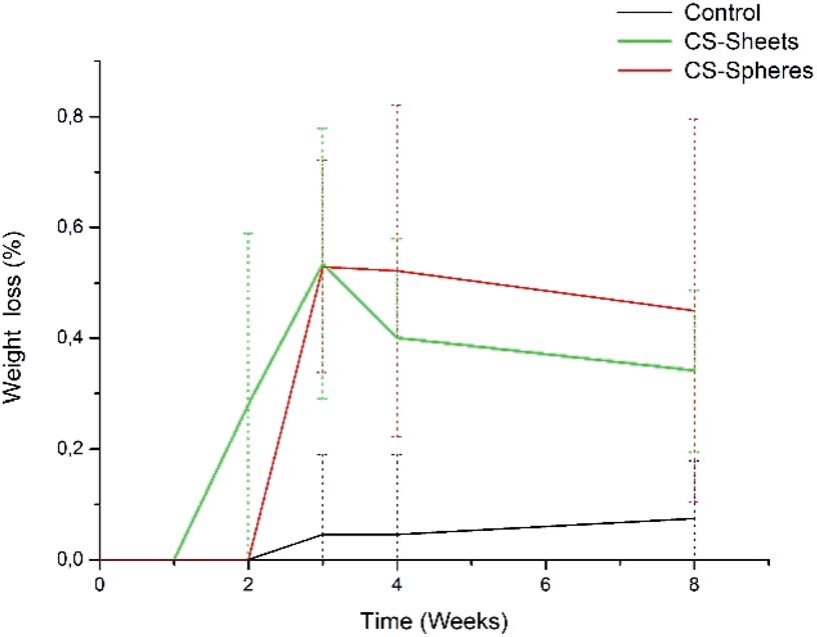
Weight loss percentage for experimental bone cements: (standard) control sample (without CS), (sheets) sample cements with 7 wt% of CS sheets, and (spheres) samples with 17 wt% of CS spheres. Collection time-points are represented by dots and standard deviation.

Analogously, Meng *et al.* reported new cements containing CS microspheres showing an increased internal macro-porosity, leading to a higher degradability of cements.^19^Fig. 4 shows the behavior of experimental cements, where the CS spheres showed a higher weight loss than cements containing CS sheets, followed by the control group. After eight weeks of immersion in SBF, the calculated values of weight loss determined for the experimental cements were: 0.07%, 0.34%, and 0.45% for control samples, CS sheets, and CS spheres, respectively. Therefore, it is expected that a higher water uptake and enhanced degradability of CS-containing cements may facilitate the creation of a network of interconnected pores and macropores, promoting cell growth and nutrient transport, degradation capacity, and bone formation within the bulk material.^17,27,31^

**Fig. 5:**
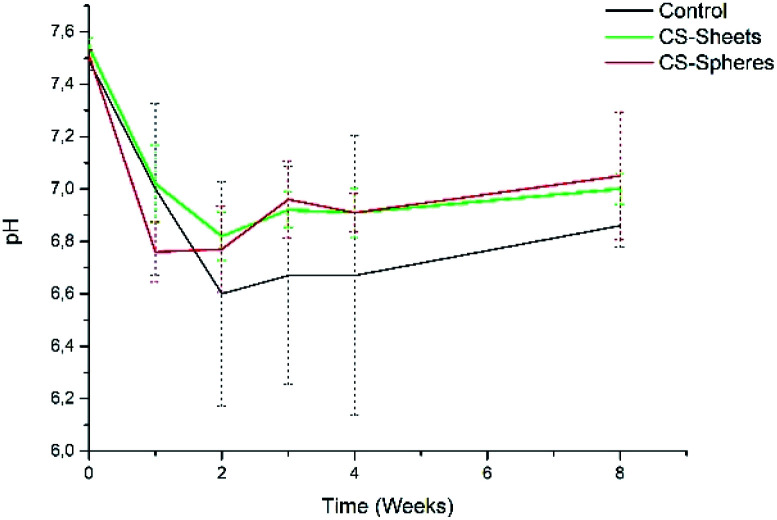
pH change of bone cements after eight weeks of introduction in SBF (control), control sample (without CS), (sheets) sample with 7 wt% of CS sheets, (spheres) sample with 17 wt% of CS spheres.

In addition, the pH measurements in hydrolytic media showed differences between experimental groups, as observed in Fig. 5. The control group presented a pH drop from 7.4 to 6.6 during the second week. From this time, the value remained relatively stable. In a similar trend, CS-containing cements also showed a drop in the pH value, reaching values of 6.8 and 6.9 for cements prepared with CS sheets and CS spheres, respectively, after two weeks. During this stage, it is expected that chito-oligomers, which are degradation products of chitosan, will be generated, such as 1,4-linked d-glucosamine and partially 1,4-linked *N*-acetyl-d-glucosamine. Studies reported that the degradation products of chitosan have significant potential due their wide bioactivity, and antibacterial and antifungal activity.^32,33^ After this time, the pH values of these three groups were slightly raised up to values of 6.7, 6.9, and 7.0, for the control, CS sheets and CS spheres cements, respectively, according to ASTM F1635 and other authors for *in vivo* implantations.^34,35^

#### Morphological analysis of bone cement after hydrolytic degradation

**Fig. 6:**
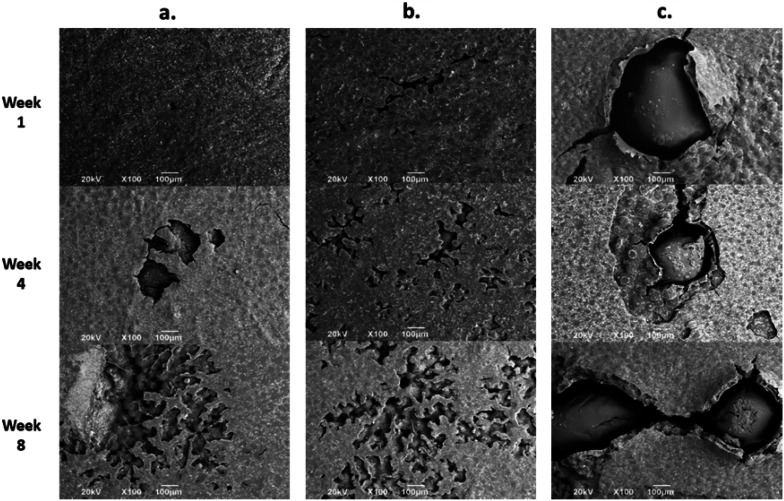
SEM micrographs at 100× of experimental bone cements immersed in SBF for 1, 4, and 8 weeks. (a) Control (without CS), (b) sample with 7 wt% of CS sheets. (c) Sample with 17 wt% of CS spheres.

Fig. 6 shows the SEM images of experimental bone cement after SBF immersion during one, four, and eight weeks of immersion. The SEM morphology of bone cement confirmed the results reported in other investigations,^17,27^ in which the incorporation of CS accelerated degradation and increased porosity from the surface to the bulk. It has been reported that this type of behavior facilitates bone integration and tissue regeneration.^19,31^

### *In vivo* biological studies

**Fig. 7:**
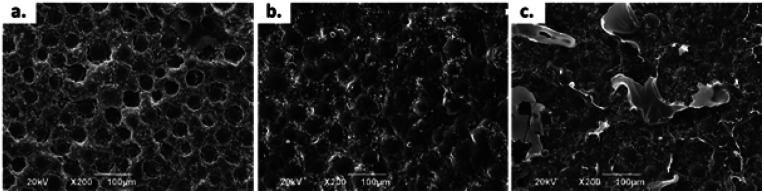
Micrographs (200×) of the surface of bone cement implanted in the subdermal region of Wistar rats after 30 days of implantation: (a) control group, (b) cement with chitosan spheres, and (c) cement with chitosan sheets.

**Fig. 8:**
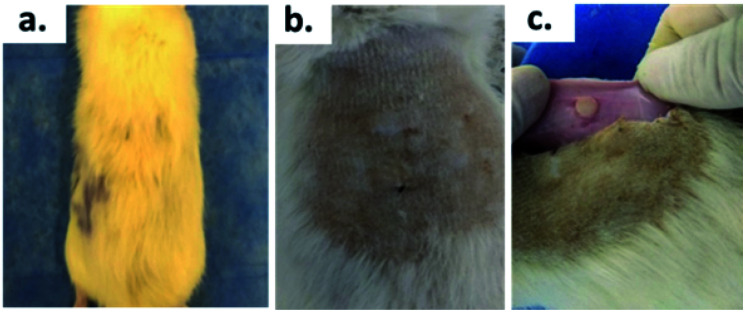
Biomodel ABC implantation: (a) with hair, (b) after the shaving process, (c) encapsulated area.

Fig. 7 shows the SEM images of the experimental bone cement implanted in subdermal tissues for 30 days. For CS-containing surfaces, it can be observed that the chitosan spheres were embedded (Fig. 8b), and the surface was rougher. This would facilitate tissue colonization once implanted in the spinal canal of the femur bone.

After 30 days of subdermal implantation, the implanted areas showed a normal appearance: during the macroscopic examination, the formation of new hair and the absence of scars and fistulous tracts were observed in the field of the incisions. On the other hand, after sample recovery, it was appreciated in the inner area of the skin that the material was encapsulated without allergic reaction or pus formation (complete healthy recover).

**Fig. 9:**
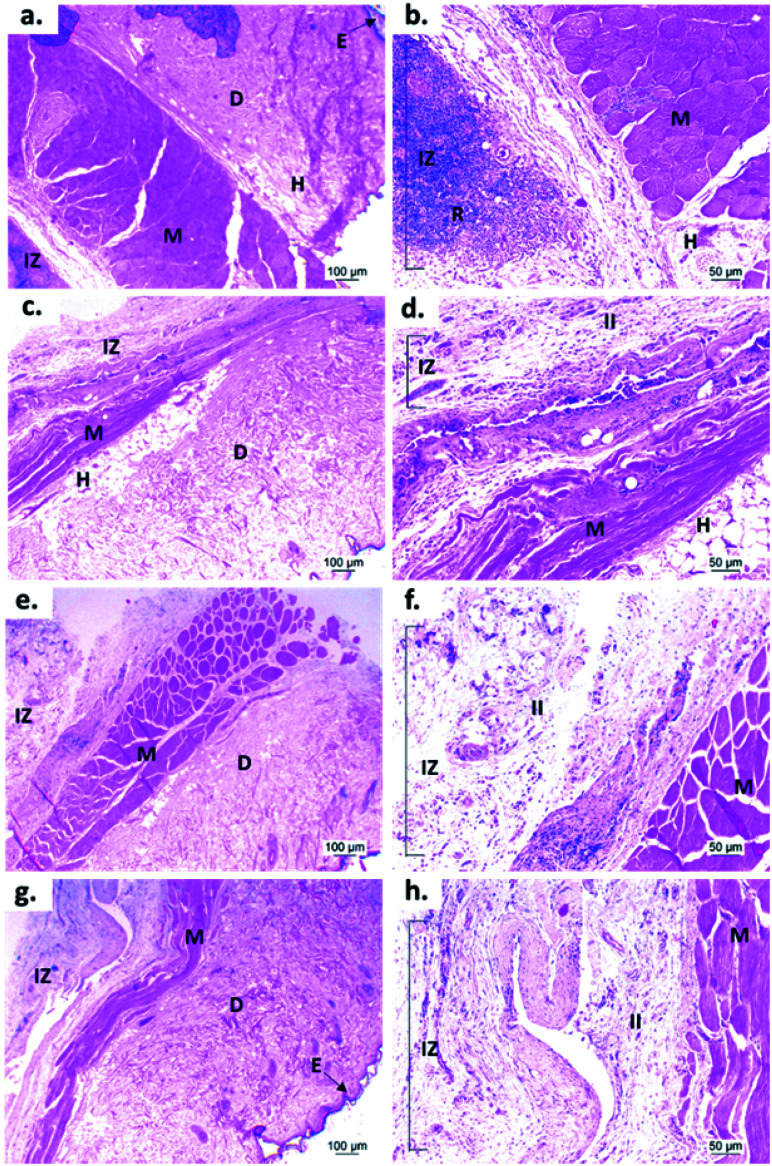
Hematoxylin and eosin (H&E) staining images of samples implanted for 30 days in the subdermal tissue of Wistar rats: (a) and (b) collagen control; (c) and (d) control cement; (e) and (f) cement with CS spheres; (g) and (h) cement with CS sheets. (a), (c), (e) and (g) 4× images; (b), (d), (f) and (h) 10× images. E: epidermis. D: dermis. M: muscle. H: hypodermis. IZ: implanted zone. FC: fibrous capsule. II: inflammatory infiltrate.

**Fig. 10:**
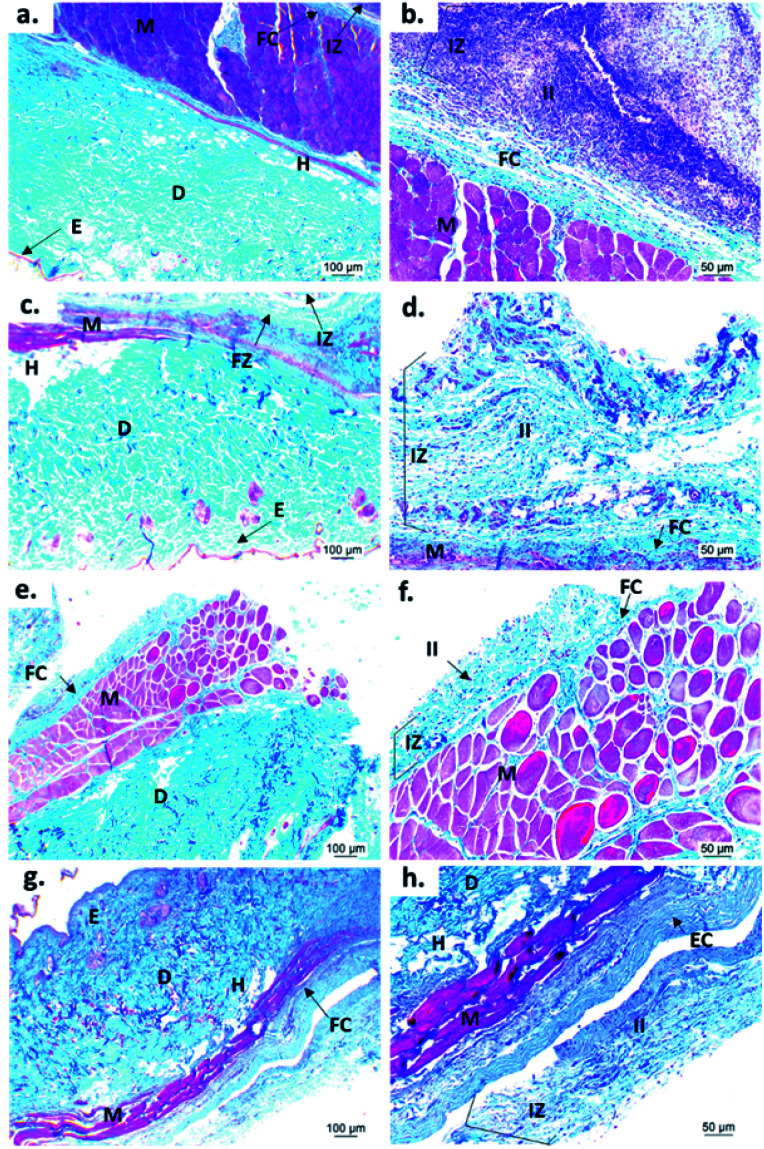
Images of Masson's trichromacy staining of cement samples implanted for 30 days in the subdermal tissue of Wistar rats: (a) and (b) collagen control; (c) and (d) control cement; (e) and (f) cement with CS spheres; (g) and (h) cement with CS sheets. (a), (c), (e) and (g) 4× images; (b), (d), (f) and h) 10× images. E: epidermis. D: dermis. M: muscle. H: hypodermis. IZ: implanted zone. FC: fibrous capsule. II: inflammatory infiltrate.

Fig. 9 shows the microscopic images of the implanted area with a soft tissue fibrous capsule surrounding the material. Collagen discs of 5 mm diameter and 2 mm thickness were inserted as the control samples. Healthy tissue architecture was observed with no remaining material. In addition, the content was not visible and the presence of abundant inflammatory infiltrate (labeled as II in Fig. 9 and 10) was found in the implantation area (labeled as IZ in Fig. 9 and 10). Similarly, the presence of inflammatory infiltrate was observed in the area of the control cement (Fig. 9c and d) and experimental cements (Fig. 9e–h), showing a higher density of inflammatory infiltrate in the area (labeled as IZ in Fig. 9 and 10), corresponding to the control material (collagen, Fig. 10b). These images support the idea that the ABCs-incorporating CS spheres and CS sheets are not negatively affected by CS introduction, and the materials are also degraded.

On the other hand, to confirm the presence of a fibrous capsule around the cements and achieve an understanding of the composition of that fibrous capsule, trichromacy Masson's stain technique was carried out (Fig. 10). In general, it is observed that all materials were surrounded by a fibrous capsule (FC) consisting mainly of type I collagen, which is a normal response due to the body's reaction to the presence of a foreign body.^36^ Remarkably, no immune responses were observed, demonstrating a biocompatibility of the cements in the subdermal tissues. This observation demonstrates that the introduction of CS spheres or CS sheets does not negatively affect the compatibility of cements, while the reabsorption process is like the collagen control.

## Conclusions

Addition of CS sheets and CS spheres demonstrated that changes in the morphology induced changes in the cement properties. Addition of higher amounts of CS spheres (17 wt%) is comparable to the addition of CS sheets (7 wt%) in compressive and flexural modulus, which could be explained for a superior contact surface for spheres. On the other hand, morphology studies demonstrated that CS sheet incorporation introduced more porosity to the cements than CS spheres. This is probably due to a better distribution of the lower size of CS spheres particles into the cement. For the hydrolytic degradation, CS introduction promoted the water absorption and degradation. Cements including CS spheres presented higher water absorption and weight loss, indicating a faster degradation process due to a bigger CS loading in the cements, as compared to CS sheets. SEM also evidenced cement porosity on the implanted samples. The high porosity degree will facilitate the cell adhesion and colonization in tissue engineering applications. H&E and Masson trichromacy stains demonstrated that cements were protected during the implantations by type I collagen tissue, and indicative of a healthy absorption and healing of the implantation process. All of the results demonstrate that controlling the CS morphology will introduce porosity and positive changes in the reabsorption process without negatively affecting the mechanical and thermal properties of cements.

## Conflicts of interest

There are no conflicts of interest to declare.

## Supplementary Material

RA-010-D0RA06508K-s001

## References

[cit1] May-Pat A., Herrera-Kao W., Cauich-Rodríguez J. V., Cervantes-Uc J. M., Flores-Gallardo S. G. (2012). Comparative study on the mechanical and fracture properties of acrylic bone cements prepared with monomers containing amine groups. J. Mech. Behav. Biomed. Mater..

[cit2] Chen L., Tang Y., Zhao K., Zha X., Liu J., Bai H., Wu Z. (2019). Fabrication of the antibiotic-releasing gelatin/PMMA bone cement. Colloids Surf., B.

[cit3] Ramakrishna S. and Huang Z.-M., Biocomposites, in Comprehensive Structural Integrity, Pergamon, 2003, pp. 215–296, ISBN 9780080437491

[cit4] Lee C. , The mechanical properties of PMMA bone cement, Well-Cemented Total Hip Arthroplast. Theory Pract, 2005, pp. 60–66

[cit5] Seyyed H. R., Emami M., Lahiji F., Shani A. S. (2016). The Acrylic Bone Cement in Arthroplasty. InTech.

[cit6] Kurtz S. M., Villarraga M. L., Zhao K., Edidin A. A. (2005). Static and fatigue mechanical behavior of bone cement with elevated barium sulfate content for treatment of vertebral compression fractures. Biomaterials.

[cit7] Lewis G. (1997). Properties of acrylic bone cement: state of the art review. J. Biomed. Mater. Res..

[cit8] Endogan T., Kiziltay A., Kose G. T., Comunoglu N., Beyzadeoglu T., Hasirci N. (2014). Acrylic Bone Cements: Effects of the Poly(methyl methacrylate) Powder Size and Chitosan Addition on Their Properties. J. Appl. Polym. Sci..

[cit9] Slane J., Vivanco J., Meyer J., Ploeg H. L., Squire M. (2014). Modification of acrylic bone cement with mesoporous silica nanoparticles: effects on mechanical, fatigue and absorption properties. J. Mech. Behav. Biomed. Mater..

[cit10] Phakatkar A. H., Shirdar M. R., Qi M., Taheri M. M., Narayanan S., Foroozan T., Sharifi-Asl S., Huang Z., Agrawal M., Lu Y. (2019). *et al.*, Novel PMMA bone cement nanocomposites containing magnesium phosphate nanosheets and hydroxyapatite nanofibers. Mater. Sci. Eng., C.

[cit11] Rojo L., Vazquez B., Deb S., San Roman J. (2009). Eugenol derivatives immobilized in auto-polymerizing formulations as an approach to avoid inhibition interferences and improve biofunctionality in dental and orthopedic cements. Acta Biomater..

[cit12] Paz E., Ballesteros Y., Abenojar J., del Real J. C., Dunne N. J. (2019). Graphene oxide and graphene reinforced PMMA bone cements: evaluation of thermal properties and biocompatibility. Materials.

[cit13] Zhao D., Yu S., Sun B., Gao S., Guo S., Zhao K. (2018). Biomedical applications of chitosan and its derivative nanoparticles. Polymers.

[cit14] Vunain E. , Mishra A. K. and Mamba B. B., Fundamentals of chitosan for biomedical applications, in Chitosan Based Biomaterials, Elsevier Inc., 2017, vol. 1, pp. 3–30, ISBN 9780081002575

[cit15] Palla-Rubio B., Araújo-Gomes N., Fernández-Gutiérrez M., Rojo L., Suay J., Gurruchaga M., Goñi I. (2019). Synthesis and characterization of silica-chitosan hybrid materials as antibacterial coatings for titanium implants. Carbohydr. Polym..

[cit16] Tan H., Ao H., Ma R., Tang T. (2013). Quaternised chitosan-loaded polymethylmethacrylate bone cement: biomechanical and histological evaluations. Journal of Orthopaedic Translation.

[cit17] Valencia Zapata M. E., Mina Hernandez J. H., Grande Tovar C. D., Valencia Llano C. H., Diaz Escobar J. A., Vázquez-Lasa B., San Román J., Rojo L., Rojo L. (2019). Novel Bioactive and Antibacterial Acrylic Bone Cement Nanocomposites Modified with Graphene Oxide and Chitosan. Int. J. Mol. Sci..

[cit18] Shi Z., Neoh K. G., Kang E. T., Wang W. (2006). Antibacterial and mechanical properties of bone cement impregnated with chitosan nanoparticles. Biomaterials.

[cit19] Meng D., Dong L., Wen Y., Xie Q. (2015). Effects of adding resorbable chitosan microspheres to calcium phosphate cements for bone regeneration. Mater. Sci. Eng., C.

[cit20] Dias F. S., Queiroz D. C., Nascimento R. F., Lima M. B. (2008). Um sistema simples para preparacao de microesferas de quitosana. Quim. Nova.

[cit21] Sharma A. K., Bhandari R., Aherwar A., Rimašauskienė R. (2019). Matrix materials used in composites: a comprehensive study. Mater. Today: Proc..

[cit22] Montgomery D. C. , Design and Analysis of Experiments, eighth edn, 2012, vol. 2, ISBN 9781118146927

[cit23] Organization I.S.-I.S. , ISO 5833, 2002, Implants for Surgery—Acrylic Resin Cements, 2002

[cit24] Kokubo T., Takadama H. (2006). How useful is SBF in predicting in vivo bone bioactivity?. Biomaterials.

[cit25] Organization I.S.-I.S. , ISO 10993-6 Biological evaluation of medical devices. Part 6: Tests for local effects after implantation, 2016

[cit26] Wahba M. I. (2020). Enhancement of the mechanical properties of chitosan. J. Biomater. Sci., Polym. Ed..

[cit27] Valencia Zapata M. E., Mina Hernandez J. H., Grande Tovar C. D. (2020). Acrylic Bone Cement Incorporated with Low Chitosan Loadings. Polymers.

[cit28] Moussout H., Ahlafi H., Aazza M., Amechrouq A. (2018). Al_2_O_3_/chitosan nanocomposite: Preparation, characterization and kinetic study of its thermal degradation. Thermochim. Acta.

[cit29] Wanis Elshereksi N., Mohamed S. H., Arifin A., Arifin Z., Ishak M. (2014). Thermal Characterisation of Poly(Methyl Methacrylate) Filled with Barium Titanate as Denture Base Material. J. Phys. Sci..

[cit30] Han J., Ma G., Nie J. (2011). A facile fabrication of porous PMMA as a potential bone substitute. Mater. Sci. Eng., C.

[cit31] Abdel-fattah W. I., Jiang T., El-Bassyouni G. E.-T., Laurencin C. T. (2007). Synthesis, characterization of chitosans and fabrication of sintered chitosan microsphere matrices for bone tissue engineering. Acta Biomater..

[cit32] Aam B. B., Heggset E. B., Norberg A. L., Sørlie M., Vårum K. M., Eijsink V. G. H. (2010). Production of chitooligosaccharides and their potential applications in medicine. Mar. Drugs.

[cit33] Logithkumar R., Keshavnarayan A., Dhivya S., Chawla A., Saravanan S., Selvamurugan N. (2016). A review of chitosan and its derivatives in bone tissue engineering. Carbohydr. Polym..

[cit34] Kim S. B., Kim Y. J., Yoon T. L., Park S. A., Cho I. H., Kim E. J., Kim I. A., Shin J.-W. (2004). The characteristics of a hydroxyapatite–chitosan–PMMA bone cement. Biomaterials.

[cit35] ASTM I. , ASTM F1635-16, Standard Test Method for in vitro Degradation Testing of Hydrolytically Degradable, 2018, pp. 1–7

[cit36] Awaja F., Tripathi M., Coraça-Huber D., Speranza G. (2018). Biocompatibility of different graphene oxide coatings on polymers. Materialia.

