# Supplementary material for: Influence of the chitosan morphology on the properties of acrylic cements and their biocompatibility
Source: RSC Adv. 2020 Aug 21;10(52):31156–64. doi: 10.1039/d0ra06508k (PMC9056417; doi:10.1039/d0ra06508k)
Supplement: RA-010-D0RA06508K-s001 [file RA-010-D0RA06508K-s001.pdf]

Electronic Supplementary Material (ESI) for RSC Advances.
This journal is © The Royal Society of Chemistry 2020

Notes

Table S1. Design of mixtures of extreme vertices for CS sheets.

Randomization	Run Order	Type	PMMA (wt. %)	CS (wt. %)	X1:Size
7	1	0	78	10	-1
9	2	1	88	0	1
2	3	1	68	20	-1
10	4	1	68	20	1
5	5	-1	73	15	-1
11	6	0	78	10	1
4	7	-1	83	5	-1
15	8	0	78	10	1
12	9	-1	83	5	1
16	10	0	78	10	1
14	11	0	78	10	1
8	12	0	78	10	-1
3	13	0	78	10	-1
6	14	0	78	10	-1
13	15	-1	0,73	0,15	1
1	16	1	0,88	0	-1

Table S2. Design of mixtures of extreme vertices for CS spheres.

Randomization	Run Order	Type	PMMA (wt.%)	CS (wt. %)
7	1	0	78	10
9	2	1	88	0
2	3	1	68	20
5	4	-1	73	15
4	5	-1	83	5
8	6	0	78	10
3	7	0	78	10
6	8	0	78	10
1	9	1	88	0

Figure S1: Optimization chart (Control-Sheets)

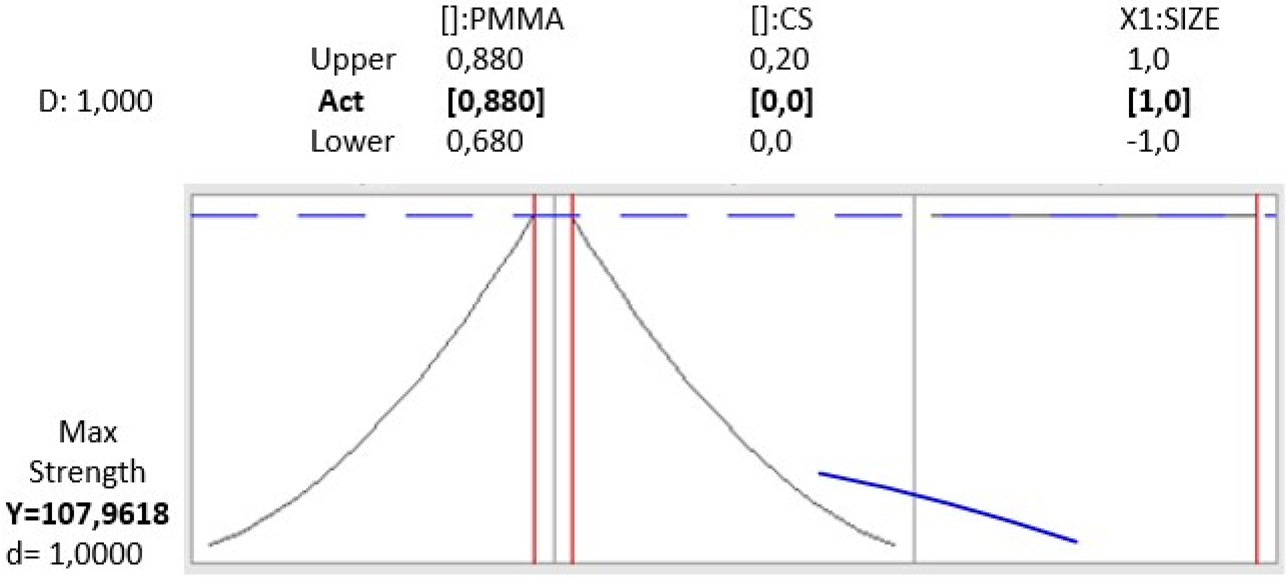

Figure S2: Optimization chart (Control-Spheres).

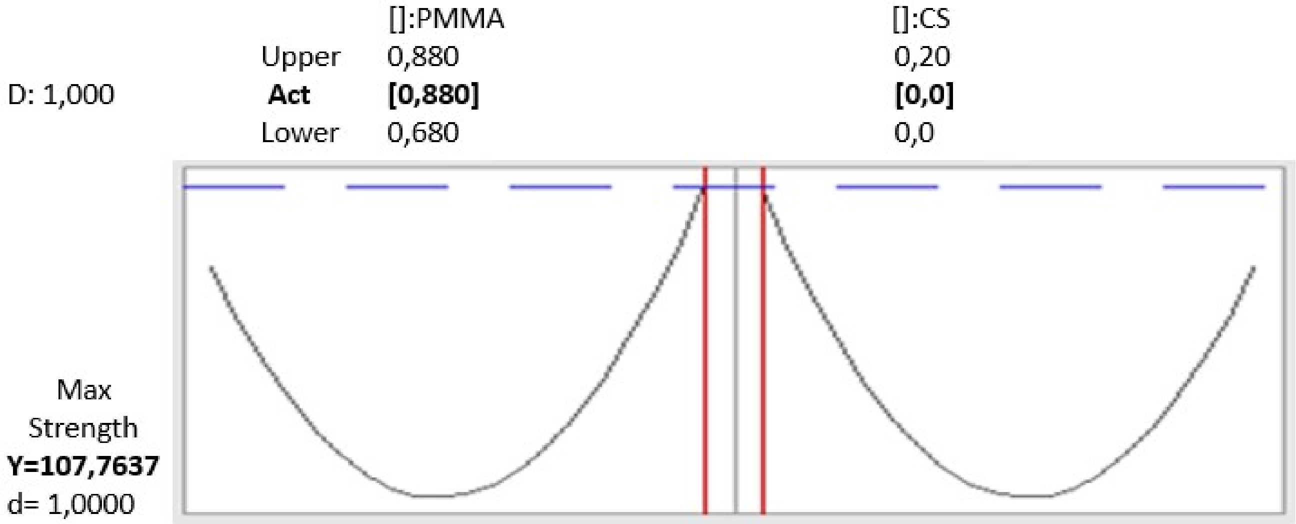

Figure S3: Optimization chart (Sheets design),

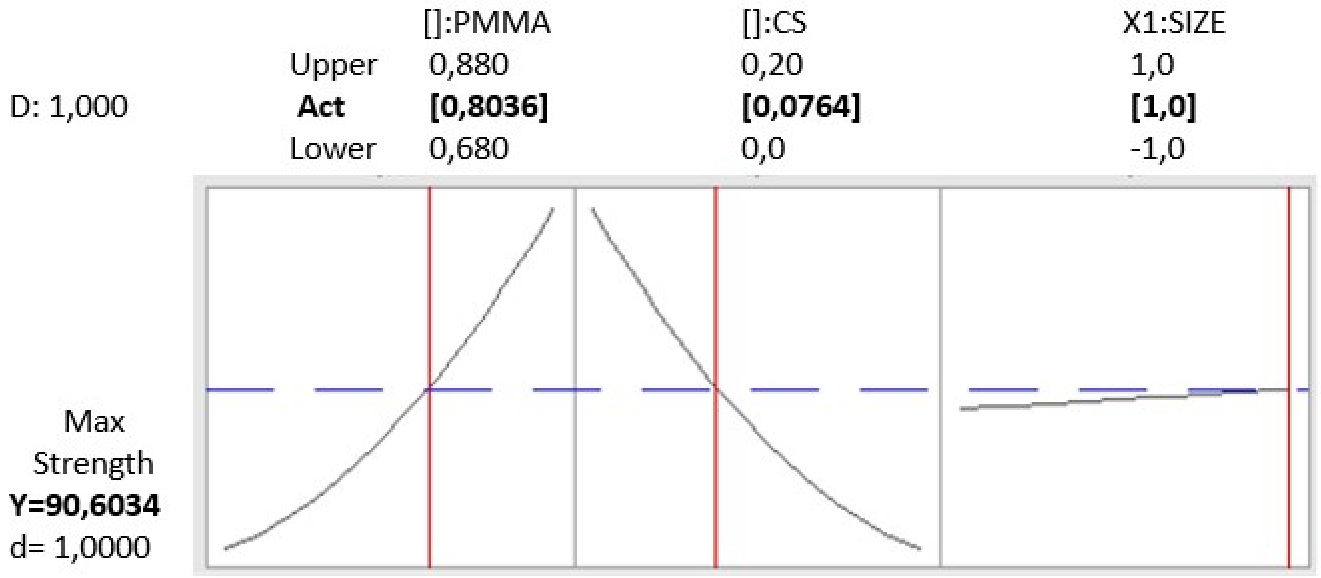

Figure S4: Optimization graphic (Spheres design).

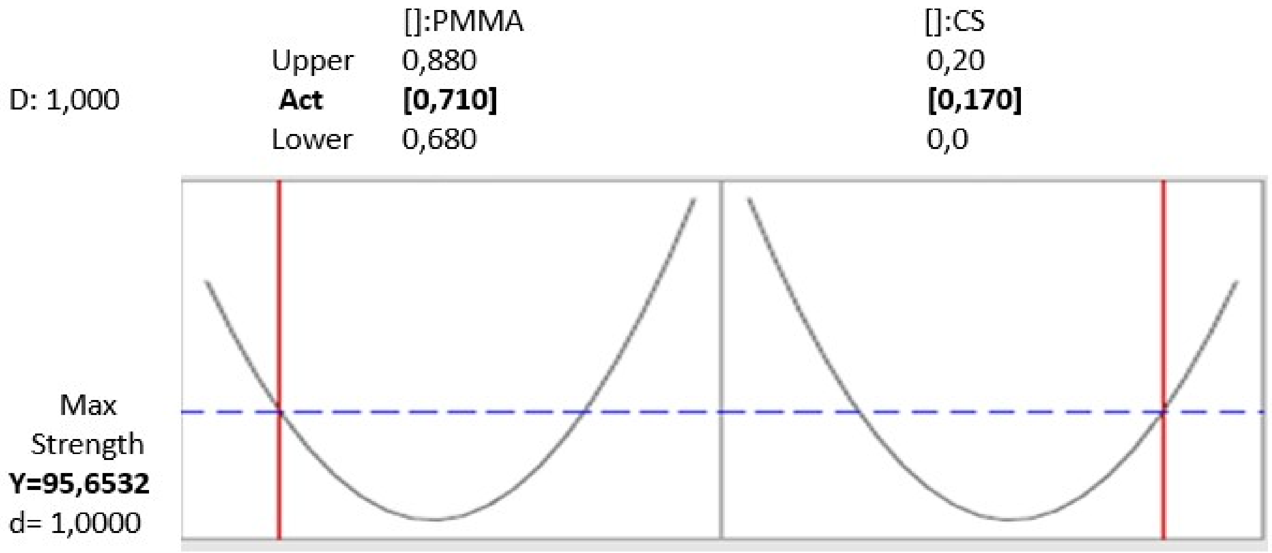

Post-curing residual monomer

Remaining residual monomer (RM) within cured cements after 24

hours were determined by ^{1}H- nuclear magnetic resonance (^{1}H-NMR, BRUKER 400 MHz. AVANCE II, United States) as calculated from equation 2. Deuterated chloroform was used as solvent and tetramethylsilane (TMS) as internal reference.

$$MRRM_{MMA}(\%) = \frac{A_{MMA}}{A_{MMA} + A_{PMMA}}(100) \qquad Equation\ 1$$

Where:

A_{MMA} is the signal area of methoxyl protons of MMA (δ=3.7 ppm).

A_{PMMA} is the signal area of methoxyl protons of PMMA (δ=3.5 ppm).

Determination of residual monomer

RM percentages of bone cement arepresented in Table 3 and it can be noted that the presence of CS in cement did not induce changes in the rm percentage showing for the three materials values are within the commonly accepted range between 2-6% for orthopaedic bone cements [17,37].

Table S3. Residual bone cement monomer according to the $^{1}H-NMR$ spectrum

Sample	Composition (wt. %)	Residual monomer (wt. %)
**Control**	88% PMMA - 0% CS	2.94
**Sheets**	80.36% PMMA – 7.64% CS	2.94
**Spheres**	71%PMMA - 17% CS	3.03
